# The interrelationships between neuronal viability, synaptic integrity, microglial responses, and amyloid-beta formation in an in vitro neurotrauma model

**DOI:** 10.1038/s41598-022-26463-w

**Published:** 2022-12-20

**Authors:** Lan-Wan Wang, Hung-Jung Lin, Chien-Ming Chao, Mao-Tsun Lin, Lin-Yu Wang, Lan-Hsiang Chein, Ching-Ping Chang, Chung-Ching Chio

**Affiliations:** 1 Department of Pediatrics, Chi Mei Medical Center, No. 901, Zhonghua Rd., Yongkang District, Tainan, 710 Taiwan; 2 Department of Biotechnology and Food Technology, Southern Taiwan University of Science and Technology, Tainan, 710 Taiwan; 3 Department of Emergency Medicine, Chi Mei Medical Center, No. 901, Zhonghua Rd., Yongkang District, Tainan, 710 Taiwan; 4 School of Medicine, Taipei Medical University, Taipei, 110 Taiwan; 5 Department of Intensive Care Medicine, Chi Mei Medical Center, Liouying, No.201, Taikang Taikang Vil., Liouying Dist., Tainan, 73657 Taiwan; 6 Department of Dental Laboratory Technology, Min-Hwei College of Health Care Management, Tainan, 73657 Taiwan; 7 Department of Medical Research, Chi Mei Medical Center, No. 901, Zhonghua Rd., Yongkang District, Tainan, 710 Taiwan; 8 Center for General Education, Southern Taiwan University of Science and Technology, Tainan, 71005 Taiwan; 9 Division of Neurosurgery, Department of Surgery, Chi Mei Medical Center, No. 901, Zhonghua Rd., Yongkang District, Tainan, 710 Taiwan

**Keywords:** Neuroscience, Cellular neuroscience, Molecular neuroscience

## Abstract

The interrelationships between neuronal viability, synaptic integrity, and microglial responses remain in infancy. In dealing with the question, we induced a stretch injury to evaluate the mechanical effects of trauma on rat primary cortical neurons and BV2 microglial cells in a transwell culture system. The viability of primary neurons and BV2 cells was determined by MTT. Synaptic integrity was evaluated by determining the expression of beta-secretase 1 (BACE1), amyloid-beta (Aβ), microtubule-associated protein 2 (MAP2), and synaptophysin (vehicle protein). Both CD16/32-positive (CD16/32^+^) and CD206-positive (CD206^+^) microglia cells were detected by immunofluorescence staining. The phagocytic ability of the BV2 cells was determined using pHrodo *E. coli* BioParticles conjugates and flow cytometry. We found that stretch injury BV2 cells caused reduced viability and synaptic abnormalities characterized by Aβ accumulation and reductions of BACE1, MAP2, and synaptophysin in primary neurons. Intact BV2 cells exhibited normal phagocytic ability and were predominantly CD206^+^ microglia cells, whereas the injured BV2 cells exhibited reduced phagocytic ability and were predominantly CD16/32^+^ microglial cells. Like a stretch injury, the injured BV2 cells can cause both reduced viability and synaptic abnormalities in primary neurons; intact BV2 cells, when cocultured with primary neurons, can protect against the stretch-injured-induced reduced viability and synaptic abnormalities in primary neurons. We conclude that CD206^+^ and CD16/32^+^ BV-2 cells can produce neuroprotective and cytotoxic effects on primary cortical neurons.

## Introduction

The interrelationships between neuronal viability, synaptic integrity, microglial responses, and amyloid-beta (Aβ) is complex, and the role of microglia response to Aβ during neurotrauma remains in infancy. Neurotrauma induces Aβ-genesis via increasing the enzymes necessary for Aβ genesis such as beta-secretase 1 (BACE1) protein and the gamma-secretase complex proteins^1^. Overexpression of proinflammatory cytokines by microglia can stimulate gamma-secretase activity and increase the production of Aβ and the intracellular domain of amyloid precursor protein (APP)^2^. Neurotrauma-induced Aβ plaques were accompanied with microglia containing Aβ, suggesting that phagocytic clearance of plaques may occur^3^ .

Microglial phagocytosis plays a vital role in brain tissue homeostasis and the response to injury. Rapid removal of dead or dying cells via microglial phagocytosis prevents the release of proinflammatory mediators and contributes to the resolution of inflammation^4^. When they encounter synaptic debris, such as accumulated amyloid-beta (Aβ), resting microglia phagocytose the synaptic debris and exert neuroprotective effects^5^. However, deficits in microglia function may contribute to synaptic abnormalities seen in some neurodevelopmental diseases^5^.

Both CD16/32-positive (CD16/32^+^) microglia cells and CD206-positive (CD206^+^) microglia cells have been promoted to possess a pro-inflammatory, tissue-damaging action and an anti-inflammatory, tissue-repairing action, respectively^6^. Generally, when the number of former anti-inflammatory, tissue-repairing CD206^+^ microglia is decreased, later CD16/32^+^ microglia predominate at the injury site at the end stage of disease^6^. However, the relationship between microglial microglial CD16/32^+^ or CD206^+^ cells and phagocytosis during neurotrauma remains unclear.

There are limited therapeutic options for TBI, which is a major cause of mortality and morbidity. In vitro stretch injury models can better reproduce clinical neuronal injury after TBI than animal models of closed head injury^7^. Neuronal injury-related neuropathological features correlate well with neurological symptoms^8^. In vitro models of stretch injury are widely used to evaluate the effects of mechanical trauma on neurons, astrocytes, and other cells^8^. Herein, we induced a stretch injury model to evaluate the mechanical effect of trauma on primary rat neurons and BV2 microglia cells in a coculture system. In a transwell coculture system, it is unsuitable for coculturing primary microglia cells with primary neurons because the culture medium inhibits microglia growth. In this study, we first explore the effects of the stretch injury on viability and synaptic integrity (e.g., genesis of Aβ, BACE1, and synaptic proteins [such as MAP2 and synaptophysin]) in primary cortical neurons. Next, we explore the effects of the stretch injury on viability and phagocytic ability in BV2 cells. Third, we explore the effects of coculturing injured BV2 cells with intact primary neurons on the viability and synaptic integrity of the neurons.

## Results

### The stretch injury-induced reduction in neuronal viability can be prevented by intact BV2 cells, whereas neuronal viability can be reduced by injured BV2 microglial cells

**Figure 1:**
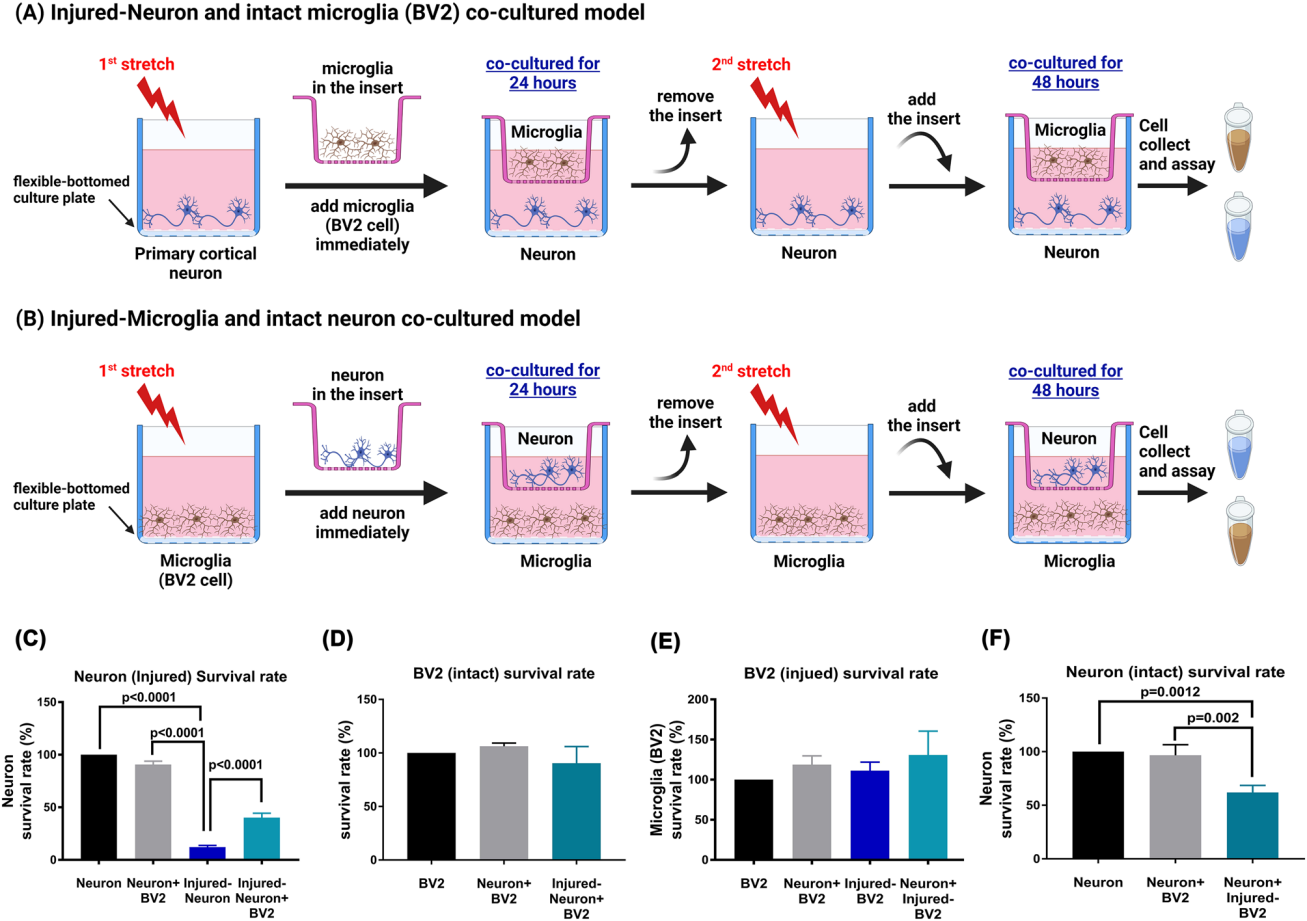
Neuronal death can be caused by stretch injury or coculture with injured BV2 microglial cells. Additionally, the stretch injury-induced reduction in neuronal viability was attenuated by coculture with BV2 cells. The rat primary cortical neurons were placed on the lower well and received 1st stretch, then cocultured with BV2 for 24 h. After 2nd stretch, we placed injured rat cortical neurons in the lower well and unchallenged BV2 cells in the insert of a Transwell coculture system for 48 h (**A**). In a separate experiment, after 2nd stretch, we placed injured BV2 cells in the lower well and unchallenged rat cortical neurons in the insert for 48 h (**B**). Measurements were made in triplicate, and each bar represents the mean ± SD. (**C**) The effects of BV2 coculture on neuron survival rate after injury [*F*(3,8) = 664.1, *P* < 0.0001]. (**D)** There was an insignificant difference in BV2 microglial survival rate between each group [*F*(2,6) = 2.271, *P* = 0.1844]. (**E**) Compared to the BV2 group, the neuron + BV2 group, the injured-BV2 group, or the neuron + injured BV2 group had an insignificant difference in microglia survival rate [*F*(3,8) = 1.773, *P* = 0.2298]. (**F**) The survival rate of neurons after coculturing with injured-BV2 [*F*(2,6) = 28.09, *P* < 0.0009]. One-way ANOVA with Tukey’s test was used for multiple comparisons.

First, a custom-built Cell Injury Controller II system was used to apply stretch injury to rat primary cortical neurons in a silastic culture plate (Flex plates) well. After 2nd stretch, we placed injured neurons in the lower well and unchallenged BV2 microglial cells in the insert of a Transwell coculture system (Fig. 1A) for 48 h. Compared to the neuron group, the injured neuron group had significantly lower cell viability (Fig. 1C). Compared to the injured neuron group, the injured neuron + BV2 cell group had significantly higher cell viability (Fig. 1C). There was an insignificant difference in BV2 microglial survival rate between the BV2 group and the neuron + BV2 group or between the BV2 group and the injured BV2 group (Fig. 1D). In a separate experiment, we placed injured BV2 cells and unchallenged cortical neurons in the lower well and insert of a Transwell coculture system, respectively (Fig. 1B). Compared to the BV2 group, the neuron + BV2 group, the injured-BV2 group, or the neuron + injured BV2 group had an insignificant difference (Fig. 1E) in microglia survival rate. However, compared to the neuron group, the neuron + injured BV2 cells, but not the neuron + BV2 group, had a significantly lower neuron survival rate (Fig. 1F).

### Stretch injury-induced axonal injury can be attenuated by intact BV2 cells, whereas synaptic integrity can be disrupted by injured BV2 cells

**Figure 2:**
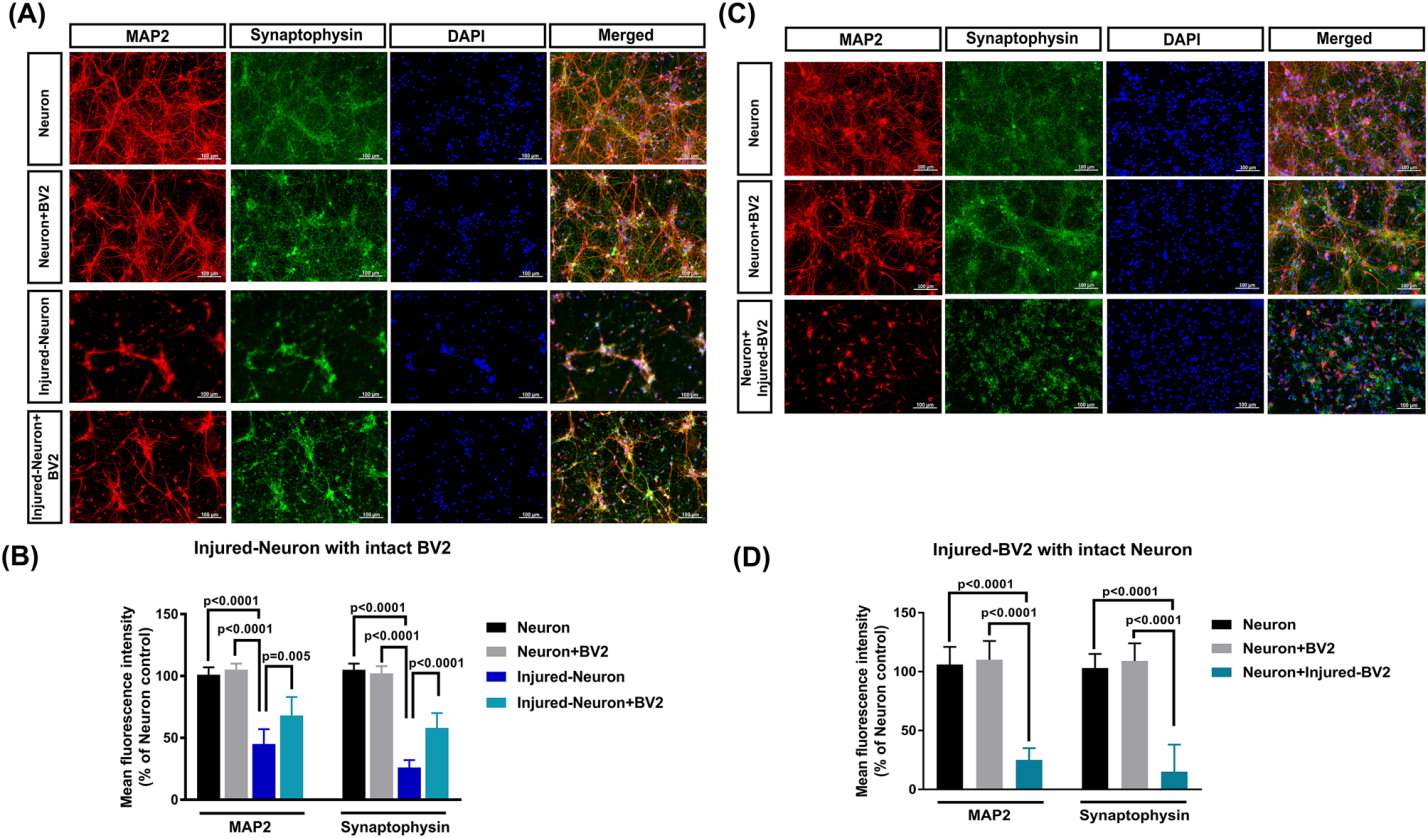
Synaptic abnormalities can be caused by stretch injury or coculture with injured BV2 microglial cells. (**A**,**C**) Representative images of immunostaining of primary rat cortical neuron cultures and the mean fluorescence intensity of MAP-2 (a microtubule-associate protein marker, red) and synaptophysin (a synaptic vesicle protein marker, green) in rat primary cortical neurons in each experimental group. All cell nuclei were counterstained with DAPI (blue). (**A**) and (**B**) depict the effects of intact BV2 cells on the altered mean fluorescence intensity of both MAP2 and synaptophysin in injured primary neurons. (**C**) and (**D**) depict the effects of injured BV2 on the mean fluorescence intensity of both MAP2 and synaptophysin in intact neurons. Each bar represents the mean ± SD of 6 independent samples per experimental condition. (**B**) There was a significant main effect for experimental groups [*F*(3,40) = 245.3, *P* < 0.0001], for MAP2 and synaptophysin [*F*(1,40) = 7.329, *P* = 0.0099], and for interaction [*F*(3,40) = 3.895, *P* = 0.0156]. (**D**) There was a significant main effect for experimental groups [*F*(2,30) = 296.4, *P* < 0.0001], but not for MAP2 and synaptophysin [*F*(1,30) = 0.9099, *P* = 0.3478] and for interaction [*F*(2,30) = 0.7232, *P* = 0.4935]. Two-way ANOVA with Tukey’s test was used for multiple comparisons.

Microtubule-associated protein 2 (MAP2) is a neuron-specific protein that promotes the assembly and stability of the microtubule network. Synaptophysin (SYP) is a synaptic vesicle protein that regulates vesicle endocytosis in neurons. Next, we asked whether the fluorescence intensity of both MAP2 and SYP in primary cultured rat cortical neurons can be affected by stretch injury. Indeed, we observed that compared to the untreated neuron group, the injured-neuron group had a significantly lower % of both MAP2 and SYP (Fig. 2A,B). However, compared to the injured-neuron group, the injured neuron + BV2 group had a significantly higher % of both MAP2 and SYP mean fluorescence intensity. In addition, compared to the neuron group, the neuron + injured-BV2 group had a significantly lower mean fluorescence intensity of both MAP2 and SYP (Fig. 2C,D).

### Stretch injury-induced neuronal Aβ accumulation can be decreased by intact BV2 cells, whereas the levels of Aβ in intact neurons can be increased by injured BV2 cells

**Figure 3:**
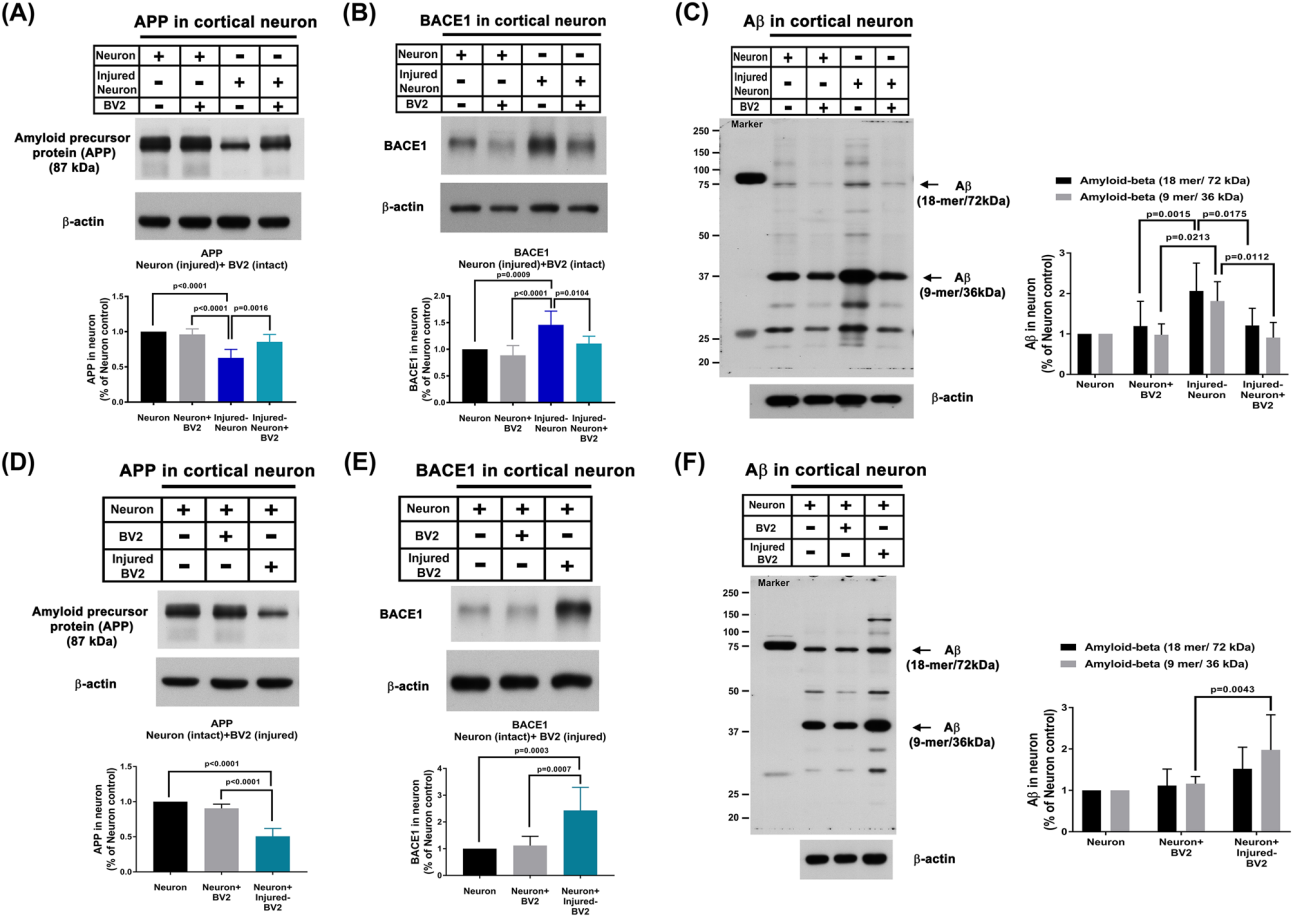
Decreased neuronal expressions of APP (**A**; [*F*(3,20) = 20.64, *P* < 0.0001]) and increased expressions of both BACE1 (**B**; [*F*(3,20) = 12.32, *P* < 0.0001]) and Aβ (**C**; group factor [*F*(3,40) = 12.8, *P* < 0.0001], Aβ factor [*F*(1,40) = 4.301, *P* = 0.0446], interaction [*F*(3,40) = 0.6942, *P* = 0.561]) in the injured-neurons can be reversed by coculture with BV2. In contrast, the neuronal levels of APP in the intact neurons were reduced by coculture with injured-BV2 (**D**; [*F*(2,18) = 88.89, *P* < 0.0001]), whereas the neuronal levels of both BACE1 (**E**; [*F*(2,18) = 15.19, *P* = 0.0001]) and Aβ (**F**; group factor [*F*(2,36) = 11.18, *P* = 0.0002], Aβ factor [*F*(1,36) = 1.463, *P* = 0.2343], interaction [*F*(2,36) = 1.118, *P* = 0.338]) were enhanced by coculture with injured-BV2. Representative immunoblots are shown. β-actin was used as a loading control. Measurements were made in triplicate, and each bar represents the mean ± SD. One-way ANOVA (for APP and BACE1 expression values) and two-way ANOVA (for Aβ expression values) with Tukey’s test were used for multiple comparisons.

As shown in Fig. 3, injured neurons had significantly lower protein levels of APP than unchallenged neurons in the neuron + BV2 cell group (Fig. 3A, for original images of the blots please see Supplementary Fig. S1) but significantly higher protein levels of both BACE1and Aβ (Fig. 3B,C, for original images of the blots please see Supplementary Figs. S2 and S3). Although intact BV2 cells did not affect the levels of APP, BACE1 or Aβ in the neurons, they significantly reversed the stretch injury-induced alteration in the protein levels of APP, BACE1, and Aβ (Fig. 3A–C). On the other hand, Fig. 3 also shows that in the coculture system, the neuronal levels of APP were significantly decreased by injured BV2 cells (Fig. 3D, for original images of the blots please see Supplementary Fig. S4). In contrast, the neuronal levels of both BACE1 and Aβ (Fig. 3E,F, for original images of the blots please see Supplementary Figs. S5 and S6) were significantly increased by coculture with injured BV2 cells. These observations suggest that in living brain tissues, some injured microglia (polarized towards the CD16/32^+^ phenotype) increase the neuronal levels of both Aβ and BACE1 but decrease the neuronal levels of APP in normal neurons, whereas uninjured microglia (polarized towards the CD206^+^ phenotype) normalize the neuronal levels of these proteins in injured neurons.

### The phagocytic capacity of BV2 microglial cells is not affected by injured neurons but is significantly reduced by stretch injury

**Figure 4:**
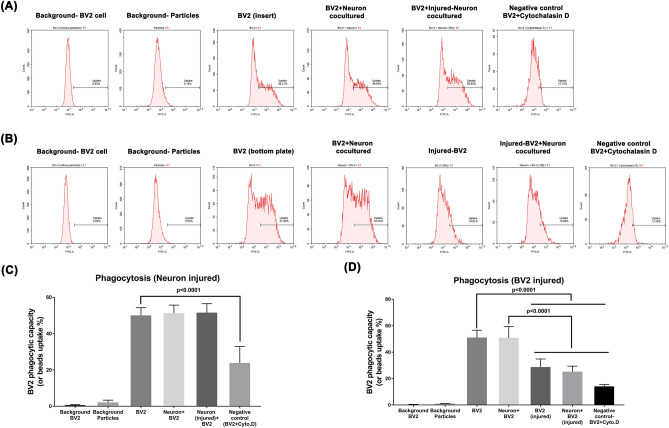
When cocultured with intact neurons or injured neurons, BV2 cells maintain their normal phagocytic capacity. The phagocytic capacity of BV2 cells was decreased by cytochalasin D or stretch injury. Representative flow cytometry plots are shown (left panels). Quantification of bead uptake (%) and BV2 cell phagocytic capacity is presented (right panels). Each bar represents the mean ± SD of 6 independent samples per experimental condition. (**C**) There was a significant difference between the Negative control (cytochalasin-treated BV2, BV2 + Cyto.D) and the BV2 group [F(5,30) = 146.8, *P* < 0.0001]. (**D**) The injured BV2 cell and neuron + injured BV2 cell groups exhibited significantly lower phagocytotic capacity than that in the BV2 cell and neuron + BV2 cell groups [*F*(6,28) = 97.1, *P* < 0.0001]. One-way ANOVA with Tukey’s test was used for multiple comparisons.

The efficiency of the BV2 microglial phagocytosis is determined by the pHrodo BioParticles Conjugates and quantified using a flow cytometer (Fig. 4A,B). Compared to the BV2 group, the neuron + BV2 group or the injured-neuron + BV2 group had an insignificant difference in BV2 phagocytic capacity (Fig. 4A,C). However, in all assays, the percentage of bead uptake by BV2 cells treated with Cytochalasin D (Cyto.D, used as a negative control to inhibit phagocytosis) was significantly lower than that by the control BV2 (23.9% vs. 50%, Fig. 4C). In a separate experiment, we found that the injured BV2 cell and neuron + injured BV2 cell groups exhibited significantly lower phagocytotic capacity than that in the BV2 cell and neuron + BV2 cell groups (Fig. 4B,D, 28.7% vs. 51% bead uptake).

### Stretch-injured BV2 cells are predominantly CD16/32^+^ cells, and unchallenged or intact BV2 microglial cells are predominantly CD206^+^ cells

**Figure 5:**
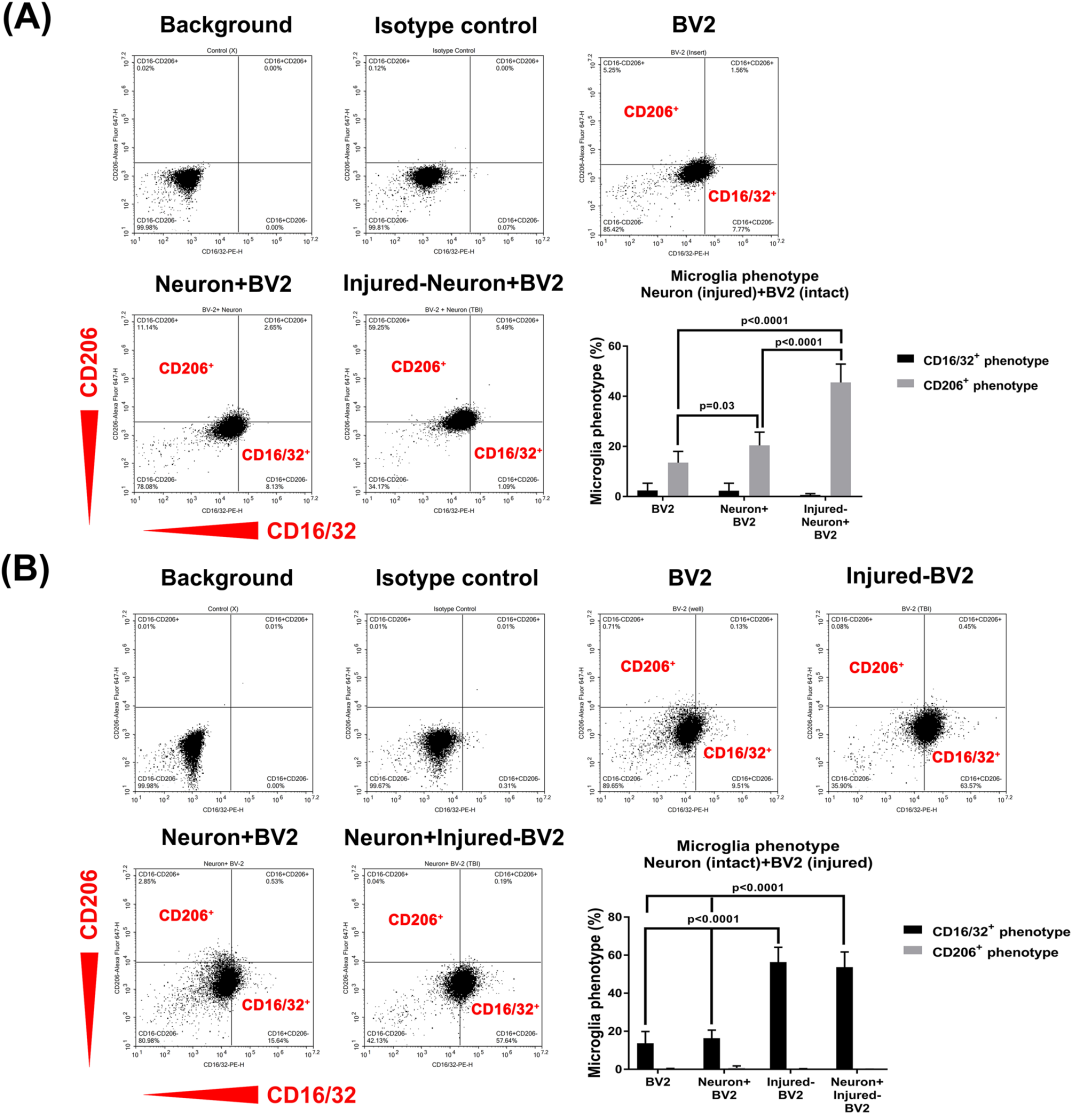
CD206^+^ microglia predominate during coculture with injured neurons, whereas CD16/32^+^ microglia predominate during stretch injury. Representative flow cytometry plots are shown (left panels). Quantification of the percentages of CD16/32^+^ microglia and CD206^+^ microglia is presented (right panels). Each bar represents the mean ± SD of 6 independent samples per experimental condition. (**A**) There was a significant main effect for group [*F*(2,30) = 37.45, *P* < 0.0001], for phenotype [*F*(1,30) = 274.1, *P* < 0.0001], and for interaction [*F*(2,30) = 47.61, *P* < 0.0001]. (**B**) There was a significant main effect for group [*F*(3,40) = 68.77, *P* < 0.0001], for phenotype [*F*(1,40) = 630.7, *P* < 0.0001], and for interaction [*F*(3,40) = 71.38, *P* < 0.0001]. Two-way ANOVA with Tukey’s test was used for multiple comparisons.

Our present study aimed to explore the effects of injured neurons and stretch-injured BV2 cells on the number of CD16/32^+^ and CD206^+^ microglia in a coculture system. We found that the percentage of cells in the injured neuron + BV2 cell group that were CD206^+^ microglia was significantly higher than that in the neuron + BV2 cell group (45.5% vs. 20.4%, Fig. 5A). However, there was no significant difference in the number of CD16/32^+^ BV2 microglia between the neuron + BV2 cell group and the injured neuron + BV2 cell group had (0.6% vs. 2.3%, Fig. 5A). Figure 5B shows that the percentage of CD16/32^+^ microglia in the injured BV2 cell group and neuron + injured BV2 cell group was significantly higher than that in the intact BV2 cell group (56.4% vs. 13.7%, 53.7% vs. 13.7%). The change in the percentage of CD16/32^+^ microglia among the different groups was negligible (Fig. 5B). As shown in Fig. 5, CD206^+^ microglia predominated during coculture with injured-neuron. However, under stretch injury, CD16/32^+^ microglial predominated (Fig. 5).

## Disscussion

At first, our results showed that moderate stretch injury significantly altered the viability of neurons but not BV2 microglial cells. Although stretch injury did not affect the viability of the BV2 microglial cells, the injured BV2 cells might cause neuronal injury by recruiting more CD16/32^+^ cells.

Next, we asked whether BV2 culture could present synaptic abnormalities following stretch injury. We found that synaptic abnormalities can be caused by stretch injury or culture with injured-BV2 cells. Additionally, stretch injury-induced synaptic abnormalities (e.g., altered microtubule and vesicle proteins) can be reversed by unchallenged BV2 cells. Again, it was found that injured-BV2 cells may cause synaptic abnormalities by recruiting more CD16/32^+^ cells, whereas unchallenged BV2 cells may preserve axonal integrity by recruiting more CD206^+^ cells.

According to a review article, Aβ is an excellent indicator of microglial response after traumatic brain injury (TBI)^9^. BACE1 (or β-secretase) and the gamma-secretase complex are associated with Aβ genesis after TBI. Our present results confirmed that injured neurons had significantly higher protein levels of both Aβ and BACE1 but significantly lower protein levels of APP than did the unchallenged neurons and cells in the neuron + BV2 cell group. Although intact BV2 cells did not affect the levels of Aβ, BACE1 or APP in the neurons, they significantly reversed the stretch injury-induced altered protein levels of Aβ, BACE1, and APP in the neurons. On the other hand, the neuronal levels of both Aβ and BACE1 were significantly increased by cocultures with injured BV2 cells, whereas the neuronal levels of APP were significantly decreased by injured-BV2 cells. These observations suggest that in living brain tissues, some injured microglia (CD16/32^+^ phenotype) might increase the levels of both Aβ and BACE1 but decrease the levels of APP in normal neurons, whereas uninjured microglia (CD206^+^ phenotype) might normalize the neuronal levels of these proteins in injured neurons.

Microglia, which resident macrophages of the central nervous system (CNS), mediate primary immune reactions^10^. Microglia phagocytose synaptic debris such as accumulated Aβ. Our present study aimed to elucidate the relationship between microglia phagocytosis and injured neurons in a culture system. Flow cytometric analysis of the phagocytic capacity of BV2 microglial cells using PHrodo *E. coli* as target particles was performed^11^. We observed that the BV2, BV2 + neuron, and BV2 + injured neuron groups exhibited a similar phagocytic capacity. On the other hand, we found that the injured BV2 cells and neuron + injured-BV2 group exhibited significantly lower phagocytotic capacity than the BV2 and neuron + BV2 groups. Our data indicate that coculturing intact or injured neurons with BV2 cellls does not affect the phagocytotic capacity of BV2 cells. However, stretch injury to BV2 cells results in disruption of phagocytosis in injured BV2 cells. Our data further showed that intact B2 cells with normal phagocytic capacity reduced the stretch injury-induced Aβ accumulation and reduced viability in primary cortical neurons. On the other hand, injured BV2 cells with reduced phagocytotic capacity caused neuronal injury and synaptic abnormalities in the cocultured primary neurons.

We used pHrodo Green *Escherichia coli* (*E. coli*) BioParticles Conjugates in the present study as a marker for phagocytic ability^12,13^. BV2 microglia were cultured, injured, and co-incubated with pHrodo-labelled E. coli over 60 min and their phagocytic and their phagocytic abilities were quantified by flow cytometry. Cytochalasin D (10 μM), an inhibitor for phagocytosis, was used as the negative control. This method assesses engulfment events but does not serve to understand the phagocytosis processing of biologically relevant cargo.

The “find-me” stage of phagocytosis, where target cells release chemotactic signals intracellularly and promote a migratory response from the phagocyte, does not occur when using BioParticles in our transwell cocultured model, nor is the “eat-me” stage which is exposed on the surface of the target cell to directly induce phagocytosis by proximal phagocytes, as BioParticles are not degraded. Therefore the last stage of phagocytosis is not assessed either. By using the *E. coli* BioParticles assay, we found that the stretch injury-induced neuronal viability and synaptic integrity reductions were attenuated by intact BV2 cells with normal phagocytic. In contrast, both the neuronal viability and synaptic integrity were reduced by coculture with injured-BV2 cells (which possess reduced phagocytic ability). Although microglia have been shown to engulf and clear damaged cellular debris after insult, deficits in microglia function may contribute to synaptic abnormalities in some neurodevelopment diseases. In our present study, microglia with normal phagocytosis may maintain neuronal viability by downregulating both Aβ accumulation and synaptic abnormalities caused by mechanical injury. In contrast, injured microglia with reduced phagocytic ability may reduce neuronal viability by upregulating both Aβ accumulation and synaptic abnormalities following mechanical injury. Therefore, we should investigate the "find-me" and "eat-me" signals in microglia and neurons under stretch stress in the future.

Our results are consistent with previous findings in models of inflammatory neurodegeneration^11,14^. Thus, inhibition of microglial phagocytosis seems sufficient to prevent neuronal death following stretch injury or inflammatory damage^14^. Microglia may improve neuronal survival by suppressing Aβ accumulation via phagocytic activity^15,16^. Microglia can engulf and clear damaged cellular debris after brain insult, wheases deficits in microglia function may contribute to synaptic abnormalities seen in some neurodevelopmental disorders^5^.

Although BV2 cells have been used frequently and widely in microglia-relevant studies, recent doubts have been raised about their suitability^17,18^. For example, a more recent study showed that BV2 cells only partially model primary microglia^19^. Thus, our present data using BV2 in microglia-related studies should be carefully considered.

The Aβ plaques found in TBI patients develop rapidly and can appear within a few hours after injury^20,21^. Additionally, Aβ accumulation after TBI is associated with an increase in BACE1 expression^22^. Microglia may clear Aβ plaques via phagocytosis during TBI^3^. Our present data show that CD206^+^ BV2 microglia possess a normal phagocytic capacity, allowing them to preserve viability and synaptic integrity in injured neurons via decreasing Aβ formation and increasing phagocytic clearance of Aβ plaques. In contrast, injured CD16/32^+^ phenotype with reduced phagocytic capacity can cause cell death and synaptic abnormalities in neurons via increasing Aβ accumulation. Our present hypothesis is consistent with several previous investigations. For example, minocycline, an inhibitor of microglial response, can treat TBI by precluding the formation of Aβ through the restoration of the nonamyloidogenic APP processing pathway involving α-secretase^23^. Other anti-inflammatory compounds also exert neuroprotective effects against TBI by enhancing the α-secretase pathway^24–26^. CD206^+^ microglia might contribute to the reduced propagation of Aβ into unaffected neurons or brain tissue^27^ via phagocytic removal of Aβ debris.

**Figure 6:**
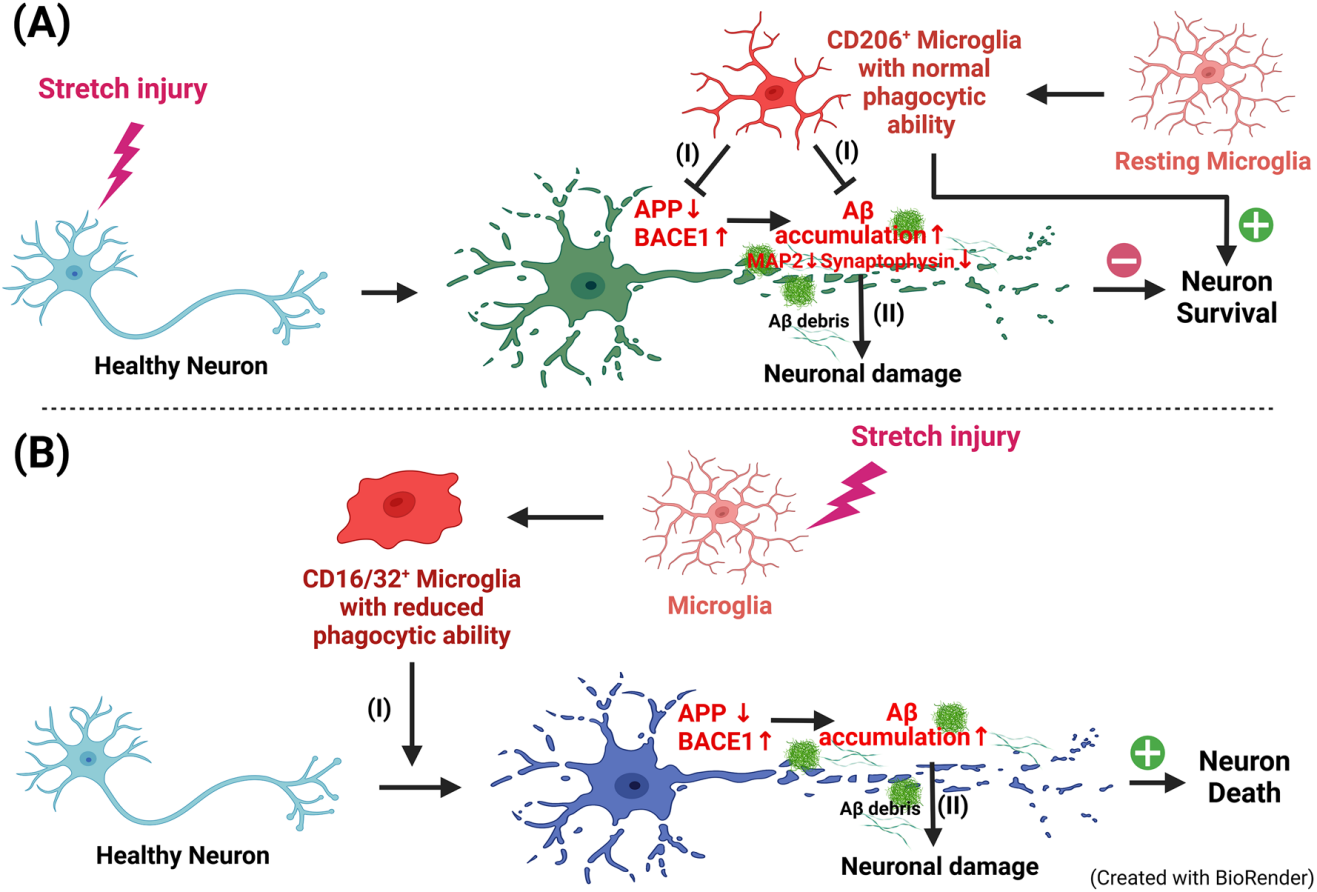
General description of the approach. (**A**) Moderate stretch injury upgrades neuronal expression of BACE1 and Aβ, downgrades neuronal expression of APP, synaptic abnormalities (e.g., decreased expression of both MAP2 and synaptophysin), and reduced viability. In addition, the injured neurons may secrete Aβ debris to injure the healthy neighborhood neurons. The CD206^+^ microglial cells with normal phagocytic capacity may protect against stretch injury via reducing synaptic abnormalities (I) and phagocytosing the Aβ debris (II). The injured microglia (CD16/32^+^) with reduced phagocytic capacity may injury the healthy neurons via upgrading neuronal expression of BACE1 and Aβ, downgrading neuronal expression of APP and downgrading the phagocytic capacity for removal of Aβ debris, and synaptic abnormalities (**B**).

As depicted in Fig. 6, our results demonstrated that moderate stretch injury causes Aβ accumulation, synaptic abnormalities, and reduced viability in rat primary cortical neurons (Fig. 6A). When cocultured with unchallenged CD206^+^ BV2 cells with normal phagocytic capacity, stretch injury-induced neuronal Aβ accumulation, axonal injury, and neuronal death are significantly reduced. Although moderate stretch injury does not significantly affect BV2 cell viability, it significantly reduces the phagocytic capacity of BV2 cells. In addition, stretch injury shifts BV2 cells from the CD206^+^ phenotype cells to the CD16/32^+^ phenotype. Accordingly, CD16/32^+^ BV2 cells with reduced phagocytic capacity cause Aβ accumulation, axonal injury, and reduced viability in cortical neurons (Fig. 6B). Microglia establish an intimate contact with Aβ plaques in the brain and become reactive^28,29^. Microglia appears activated in the vicinity of Aβ plagues. Microglia contribute to the propagation of Aβ into affected brain tissue^27^. In the present results, the CD206^+^ BV2 cells and CD16/32^+^ cells might delay and accelerate the propagation of Aβ into the unaffected primary cortical neurons, respectively, and result in neuroprotection and cytotoxic effects (Fig. 6AII,BII). To understand microglia functions and the underlying signaling machinery, many attempts were made to employ functional in vitro studies of microglia. The range of available cell culture models is broad, and they come with different advantages and disadvantages for functional assays^30^. They discuss the potentials and shortcomings of transformed cell lines (e.g., BV2 cells) and coculture models for functional studies in vitro. Although our present data indicate that BV2 microglia may improve neuronal survival in stretch injured primary cortical neurons by suppressing Aβ accumulation via maintaining their engulf ability, future experiments examining the interaction between coculturing neurons with other damaged cells should be performed to get more information about the specific effect of damaged BV2.

## Conclusion

Using a microglia-neuron cocultured system, we found that stretch injury-induced amyloid-beta (Aβ) accumulation, axonal injury, and reduced viability in cultured primary rat cortical neurons in vitro. Intact and injured microglia were predominatly CD206^+^ microglia and CD16/32^+^ microglia, respectively. Stretch injury or injured BV2 cells caused Aβ accumulation, synaptic abnormalities, and reduced viability in cortical neurons; however, intact BV2 cells protected against stretch-induced injury. Our data suggest that recruitments of more CD206^+^ or CD16/32^+^ cells can produce neuroprotective and cytotoxic effects, respectively, on primary cortical neurons.

## Materials and methods

### Ethical approval

Primary cortical cells were prepared from pregnant Wistar rats obtained from BioLASCO Taiwan Co., Ltd. at embryonic day 18 (ED18). The Institutional Animal Care and Use Committee of Chi Mei Medical Center (IACUC approval no. 106121110) approved all animal experiments. All experiments were performed in accordance with the National Institutes of Health Guide for the Care and Use of Laboratory Animals. The study was conducted in accordance with ARRIVE guidelines^31^.

### Primary cortical neuron culture

As described previously^32^, the primary cortical neurons were obtained fromWistar rat fetuses at ED18 and maintained for 8 to 10 days before the experiments. Cerebral cortices were isolated and dissociated by Papain (40 mg/ml) (#5125, Sigma- Aldrich, MO, USA) digestion and trituration with a 10 ml plastic pipette. Primary cortical neurons were suspended in a Neurobasal medium (#21103049, GIBCO/Life Technologies, NY, USA) supplemented with 2% B27 (#17504044, GIBCO/Life Technologies), 100 U/ml penicillin, 100 μg/ml streptomycin (# 15140-122, GIBCO/Life Technologies) and 200 mM GlutaMAX™ Supplement (# 35050-061, GIBCO/Life Technologies). Neurons were plated at a density of 3 × 10^6^/well in 6-well plates (#BF-3001C, FLEXCELL, NC, USA) pre-coated with Poly-L-Lysine (#3438-100-01, R&D Systems, Minneapolis, MN, USA) (0.1 mg/ml). Neurons were cultured up to 7 days in vitro (7 DIV) at 37 °C in a humidified 5% CO_2_ incubator. Half the medium was changed every 4 days. The cells were plated at a high density (3 × 10^6^ cells/well) on a poly-l-lysine-coated well and cultured in a neurobasal medium (Invitrogen).

### Stretch injury model of TBI^33^

To mimic in vivo brain injury, cultured primary monolayer cortical neurons were subjected to stretch injury via a Cell Injury Controller II (Custom Design and Fabrication; Virginia Commonwealth University). Briefly, the wells of a flexible-bottomed culture plate (Flexcell Co., Burlington, NC, USA) were sealed with a plug connected via tubing to a nitrogen tank with a pressure controller. Application of two 99 ms pluses (30–35 psi) resulted in a peak pressure of 3.5 psi in the well, which caused a moderate stretch injury with a center deflection of 10.5 mm.

### BV2 microglial cell culture

BV2 microglial cells were obtained from the American Type Culture Collection (ATCC, Manasses, VA, USA). BV2 cells were cultured in RPMI1640 (#31800-022, GIBCO/Life Technologies) supplemented with 10% fetal bovine serum (# 26140-079, GIBCO/Life Technologies), 100 U/ml penicillin, 100 μg/ml streptomycin (# 15140-122, GIBCO/Life Technologies) and 200 mM GlutaMAX™ Supplement (# 35050-061, GIBCO/Life Technologies). The cells were plated at a density of 2 × 10^6^ cells/insert.

### Coculture of primary cortical neurons and BV2 microglial cells

We placed unchallenged rat cortical neurons (3 × 10^6^) in the lower well (neuron group), neurons in the lower well and BV2 microglial cells (2 × 10^6^) on the insert (BV2 cell + neuron group), injured neurons in the lower well (injured neuron group), or injured neurons in the lower well and BV2 microglial cells in the insert (BV2 cell + injured neuron group) of a Transwell coculture system (Fig. 1A). Forty-eight hours after coculturing, neurons were collected to assess cell viability, the number of synaptophysin-expression neurons, and protein levels. In addition, BV2 microglial cells were collected to evaluate phagocytosis and phenotype.

### Cell survival assay

The viability of primary cortical neurons and BV2 microglial cells was determined using 3-(4,5-dimethyl-thiazol-2-yl)-2,5-diphenyltetrazolium bromide (MTT;#298931, Serva Electrophoresis GmbH, Heidelberg, Germany). After coculture, neurons were treated with MTT solution, and the absorbance of the sample was read at 570–630 nm with a spectrophotometer (Thermo Fisher Scientific, MultiSkan GO, Waltham, MS, USA).

### Immunofluorescence analysis of synaptophysin-expressing neurons

Cortical neurons were seeded on sterile poly-l-lysine coverslips placed in 6-well culture plates and incubated with mouse microtubule-associated protein-2 (MAP2; 1:100, #SC-74421, Santa Cruz Biotechnology Inc., Texas, USA) and rabbit synaptophysin (1:250, #MA5-14532, Thermo Fisher, MA, USA) antibodies at 4 °C overnight. Then the cells were incubated with appropriate secondary antibodies (Alexa Fluor 568 or Alexa Fluor-488-conjugated goat anti-mouse or anti-rabbit IgG secondary antibodies; 1:400, #A11008 and #A11004, Invitrogen/Life Technologies) at room temperature for 1 h. The slides were subsequently washed with phosphate buffer, and the nuclei were co-stained with 4,6-diamidino-2-phenylindole (DAPI; 1:5000, #D1306 Invitrogen) using 4′,6-diamidino-2-phenylindole (DAPI)-staining mounting medium (Vectashield Vector Laboratories, Burlingame, CA, USA). Glass coverslips were mounted on the slides using mounting medium. Digital images were captured with a 40× objective (numerical aperture (N .A.) 0.75) and a 100× oil immersion objective (NA 1.4) by an upright fluorescence microscope system (Carl Zeiss Microscopy GmbH, Jena, Germany) with Zen Software (Carl Zeiss). MAP2- and synaptophysin-positive cells were counted. The data are presented as the percentage of synaptophysin-expressing neurons in four fields per coverslip and six coverslips per experimental condition. All images were converted to grayscale to quantify fluorescence intensity and analyzed using ImageJ software. A fixed threshold range of 10–150 was chosen to highlight the fluorescence signals.

### Analysis of phagocytosis by BV2 microglia cells

The pH-sensitive green fluorophore-tagged *Escherichia coli* (*E. coli*) bioparticles (pHrodo Green *E. coli* BioParticles Conguates, # P35366, Invitrogen Corporation, Frederick, MD, USA) were used to measure the phagocytosis activity. The *E. coli* bioparticles are nonfluorescent at neutral pH. When the microglia engulf the bioparticle, phagocytic cargo presents early phagosomes of higher pH, which sequentially mature into late phagosomes, becoming increasingly acidic and finally fusing with the lysosomes for degradation and clearance. Briefly, BV2 microglial cells (5 × 10^5^/well) were plated in 6-well plates and cultured for 24 to 72 h. A total of 100 μg of pHrodo Green *Escherichia coli* (*E. coli*) BioParticles Conguates were added per condition and incubated with BV2 cells for 30 min at 37 °C. Phagocytosis was inhibited with 10 μM cytochalasin D (Cyto. D; #SI-C8273, Sigma-Aldrich), which was added 30 min before the addition of PHrodo *E. coli* bioparticles as a negative control. Cells were gated based on forward scatter (FSC)/side scatter (SSC) properties by Novocyte flow cytometry (ACEA Biosciences, CA, USA). Then adjusted threshold to eliminate debris, and the percentage of the pHrodo + cell was determined.

### Analysis of microglia phenotype

BV-2 cells in the culture insert were digested using trypsin and then washed and re-suspended in cold PBS. The cells were fixed with Fix/Perm working solution (Transcription Factor Buffer Set, #562574, BD Pharmingen, CA, USA) for 1 h on ice. The cells were incubated with PE-conjugated CD16/32 antibody (1:200, #561727, BD Biosciences) and Alexa Fluor 647-conjugated CD206 antibody (1:200, #56250, BD Biosciences) for 45 min. The isotype of the CD16/32 antibody or CD206 antibody was used as the negative control. Cells were gated based on FSC/SSC and analyzed using the excitation and emission maximum of approximately 496 and 576 nm (PE-conjugated CD16/32) or 653 and 669 nm (Alexa Fluor 647-conjugated CD206) by Novocyte flow cytometry (ACEA Biosciences, CA, USA).

### Protein extraction and Western blot analysis

Cells were lysed by shaking in radioimmunoprecipitation assay (RIPA) lysis buffer. The protein concentration was measured by the Protein assay Dye Reagent Concentrate (#5000006, Bio-Rad Laboratories, CA, USA). Cell extract (30 µg) was loaded to a sodium dodecyl sulfate (SDS)-polyacrylamide gel electrophoresis, transferred to a polyvinylidene fluoride (PVDF, #GE10600023, Merck KGaA, Darmstadt, Germany) membrane, and then blocked with PBST containing 5% skimmed milk for 1 h. The membrane was incubated with a rabbit polyclonal antibodies against amyloid precursor protein (APP; 1:30,000, #ab32136, Abcam), beta-secretase 1 (BACE1; 1: 4000, #ab2077, Abcam), Aβ (1:8000, #ab62658, Abcam), and β-actin (1:5000, #Sc-4778, Santa Cruz). On the next day, the membrane was washed four times for 15 min each with PBST and incubated with horseradish peroxidase-conjugated anti-rabbit IgG (1:20,000–100,000, #7074, Cell Signaling) or mouse IgG (1:5000, #7076S, Cell Signaling) for 1 h. The protein bands were detected for 1 min by Western Lightning ECL Pro Enhanced Luminol Reagent (#ORT2405 & #ORT2505, PerkinElmer, MA, USA) and exposed to X-ray film. The films were scanned, and the signal densities were quantified using TotalLab v2.01 software. The level of each protein was determined after normalization to the β-actin level.

### Statistics

The data are presented as the mean ± SD. Statistical analyses were performed using GraphPad Prism (version 7.04 for Windows; GraphPad Software, San Diego, CA, USA). Data matrices were first tested for normality and homoscedasticity with Shapiro's Wilk and Levene's test. The following parametric tests were applied according to the data characteristics and when the required data assumptions were fulfilled. Cell viability, APP and BACE1 expression, and phagocytosis were analyzed by the one-way ANOVA followed by Tukey’s post hoc test. Synaptic plasticity, Aβ expression, and microglia phenotypes were analyzed by the two-way ANOVA followed by Tukey’s post hoc test. The significant level was set at *P* < 0.05.

## Supplementary Information

Supplementary Figures.

## Data Availability

The datasets used and/or analyzed during the current study are available from the corresponding author on reasonable request.

## Abbreviations

AD: Alzheimer’s disease
APP: Amyloid precursor protein
Aβ: Amyloid beta
BACE1: Beta secretase 1
CTE: Chronic traumatic encephalopathy
Cyto.D: Cytochalasin D
MAP2: Microtubule-associated protein-2
MTT: 3-(4,5-Dimethyl-thiazol-2-yl)-2,5-diphenyltetrazolium bromide
TBI: Traumatic brain injury
