# Supplementary material for: The interrelationships between neuronal viability, synaptic integrity, microglial responses, and amyloid-beta formation in an in vitro neurotrauma model
Source: Sci Rep. 2022 Dec 20;12:22028. doi: 10.1038/s41598-022-26463-w (PMC9768168; doi:10.1038/s41598-022-26463-w)
Supplement: Supplementary file 1 — Supplementary Figures. [file 41598_2022_26463_MOESM1_ESM.docx]

**Supplementary data**

**The interrelationships between neuronal viability, synaptic integrity, microglial responses, and amyloid-beta formation in an *in vitro* neurotrauma model**

Lan-Wan Wang^1,2,^†, Hung-Jung Lin^3,4,^†, Chien-Ming Chao^5,6^, Mao-Tsun Lin^7^, Lin-Yu Wang^3,8^, Lan-Hsiang Chein^7^, Ching-Ping Chang^7,^*, Chung-Ching Chio^9,^*

^1^ Department of Pediatrics, Chi Mei Medical Center, Tainan 710, Taiwan

^2^ Department of Biotechnology and Food Technology, Southern Taiwan University

of Science and Technology, Tainan 710, Taiwan

^3^ Department of Emergency Medicine, Chi Mei Medical Center, Tainan 710,

Taiwan

^4^ School of Medicine, Taipei Medical University, Taipei 110, Taiwan

^5^Department of Intensive Care Medicine, Chi Mei Medical Center, Liouying, Tainan 73657, Taiwan.

^6^Department of Dental Laboratory Technology, Min-Hwei College of Health Care Management, Tainan 73657, Taiwan.

^7^ Department of Medical Research, Chi Mei Medical Center, Tainan 710, Taiwan

^8^ Center for General Education, Southern Taiwan University of Science and

Technology, Tainan City 71005, Taiwan

^9^ Division of Neurosurgery, Department of Surgery, Chi Mei Medical Center,

Tainan 710, Taiwan

†These authors contributed equally to this work.

**Correspondence:**

**Ching-Ping Chang, Ph.D.**

Department of Medical Research, Chi Mei Medical Center, Tainan, Taiwan.

Add: No. 901, Zhonghua Rd., Yongkang District, Tainan City 710, Taiwan

Email: [jessica.cpchang@gmail.com](mailto:jessica.cpchang@gmail.com) or [a50831@mail.chimei.org.tw](mailto:a50831@mail.chimei.org.tw)

**Chung-Ching Chio, MD.**

Division of Neurosurgery, Department of Surgery, Chi Mei Medical Center; No. 901, Zhonghua Rd, Yongkang District, Tainan City 710, Taiwan.

Email: [chiocc@ms28.hinet.net](mailto:chiocc@ms28.hinet.net)

**
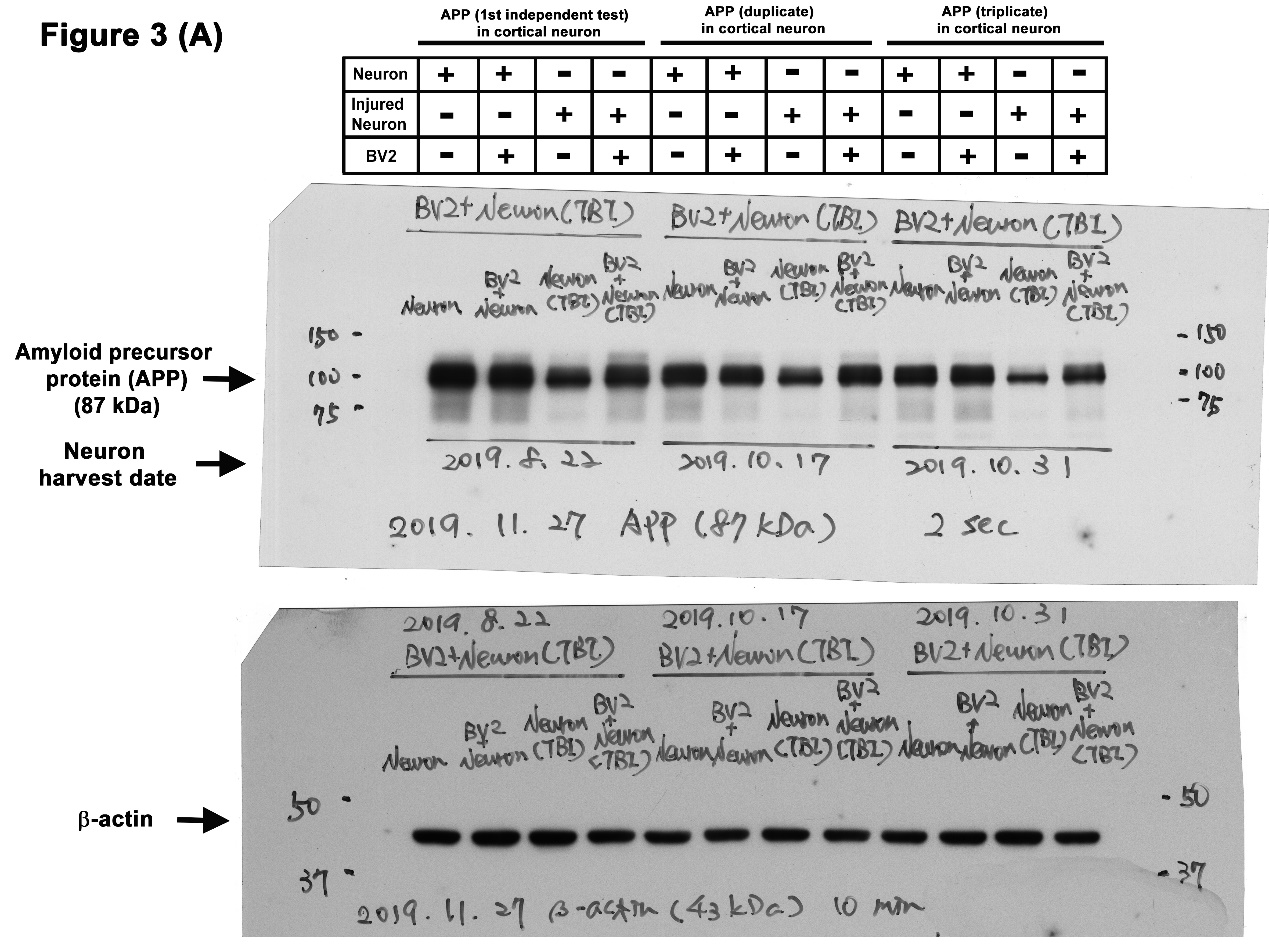
**

**Supplementary Figure S1:** Original western blots for Figure 3 (A). Labels on the right of each x-film scan indicate the antibody used for detection. Dashed red boxes show the regions that were cropped for the figures.


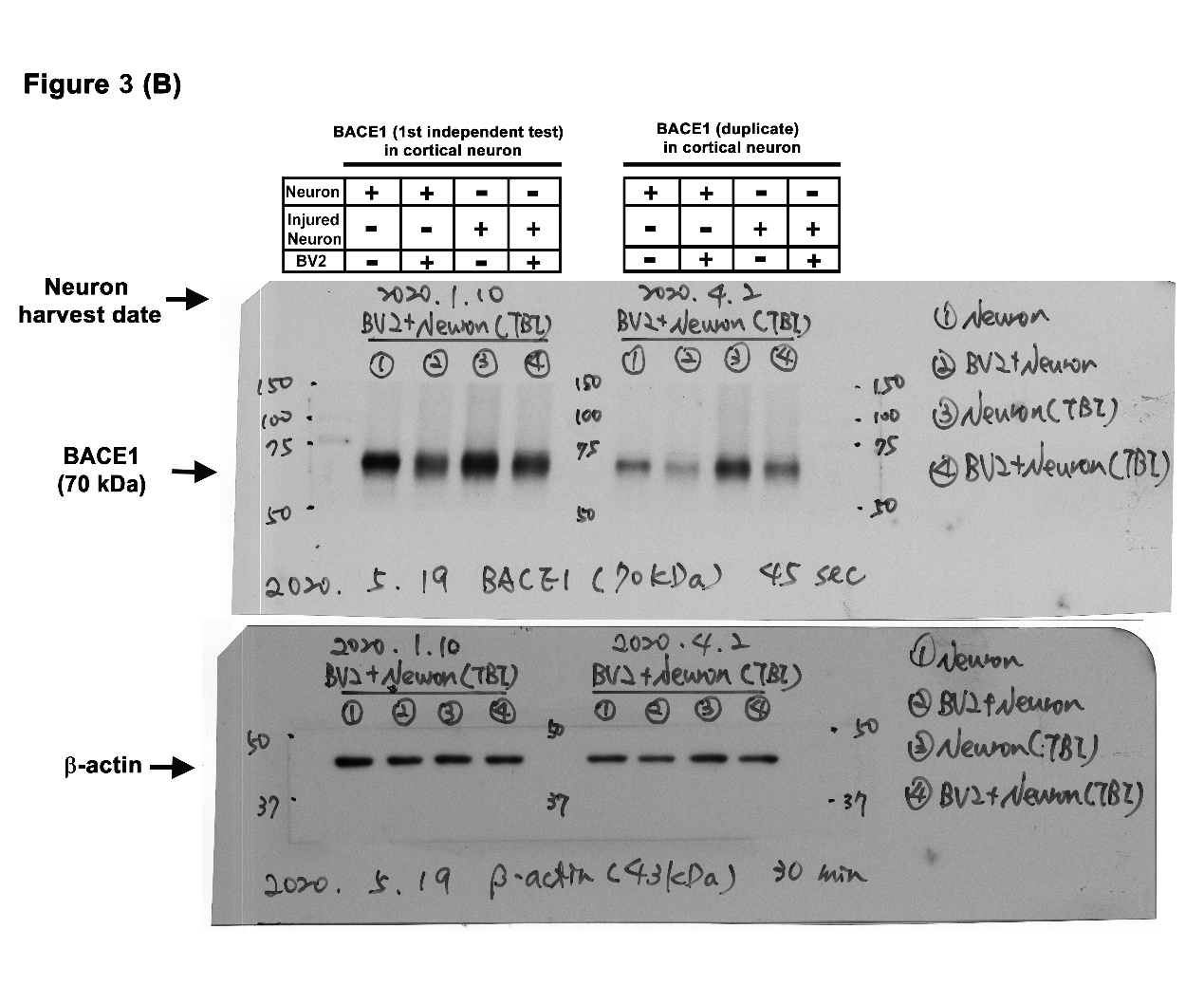


**Supplementary Figure S2:** Original western blots for Figure 3 (B). Labels on the right of each x-film scan indicate the antibody used for detection. Dashed red boxes show the regions that were cropped for the figures.


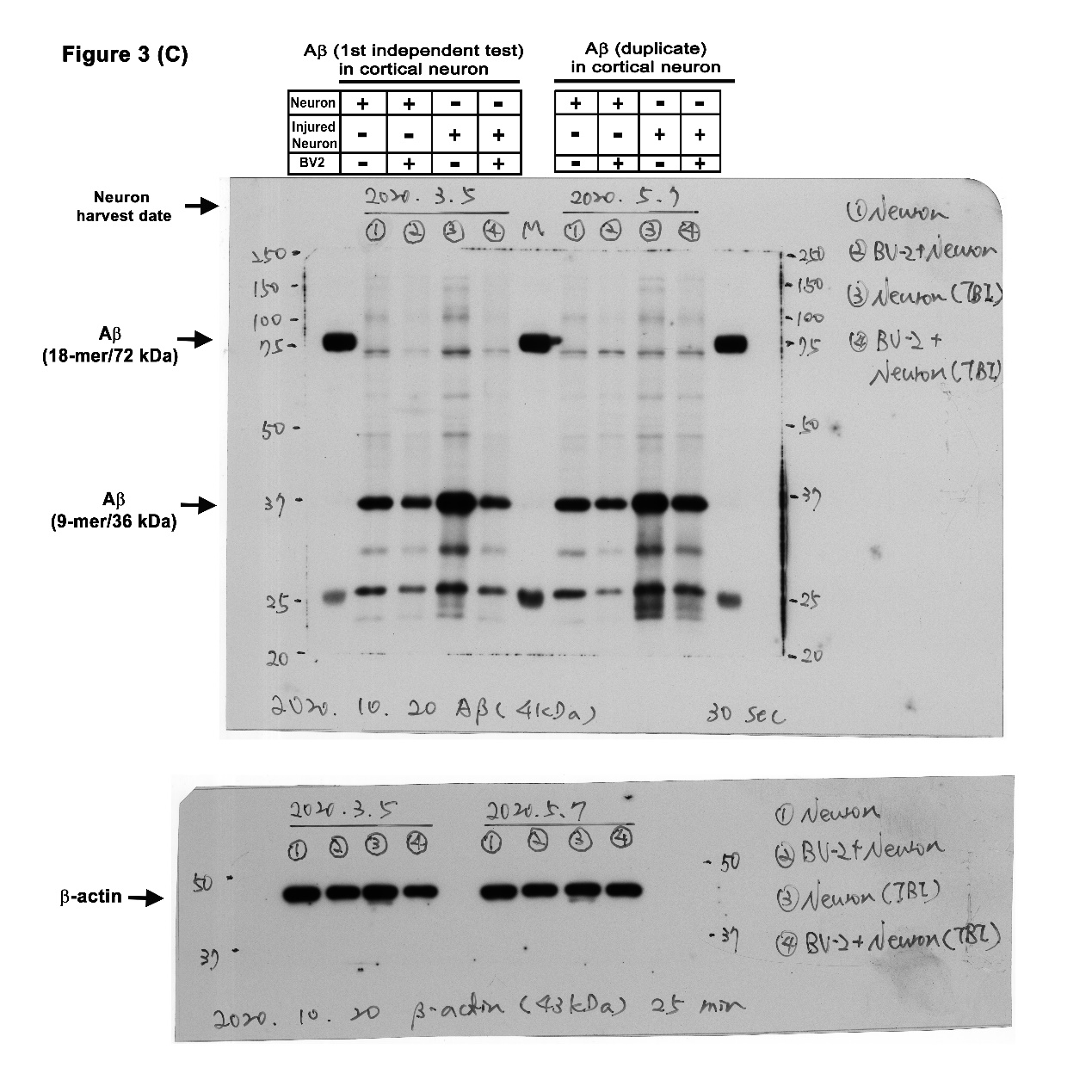


**Supplementary Figure S3:** Original western blots for Figure 3 (C). Labels on the right of each x-film scan indicate the antibody used for detection. Dashed red boxes show the regions that were cropped for the figures.


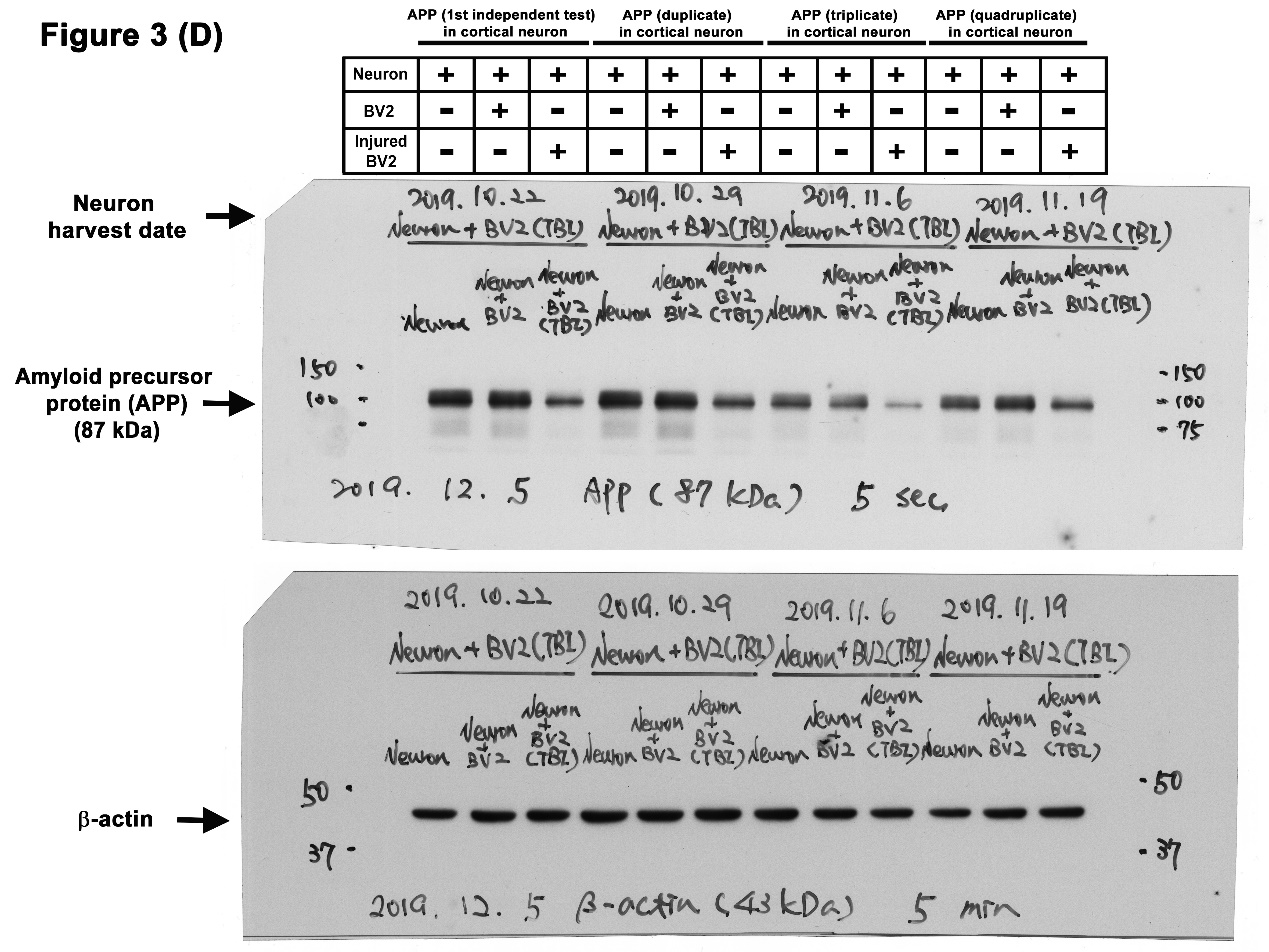


**Supplementary Figure S4:** Original western blots for Figure 3 (D). Labels on the right of each x-film scan indicate the antibody used for detection. Dashed red boxes show the regions that were cropped for the figures.


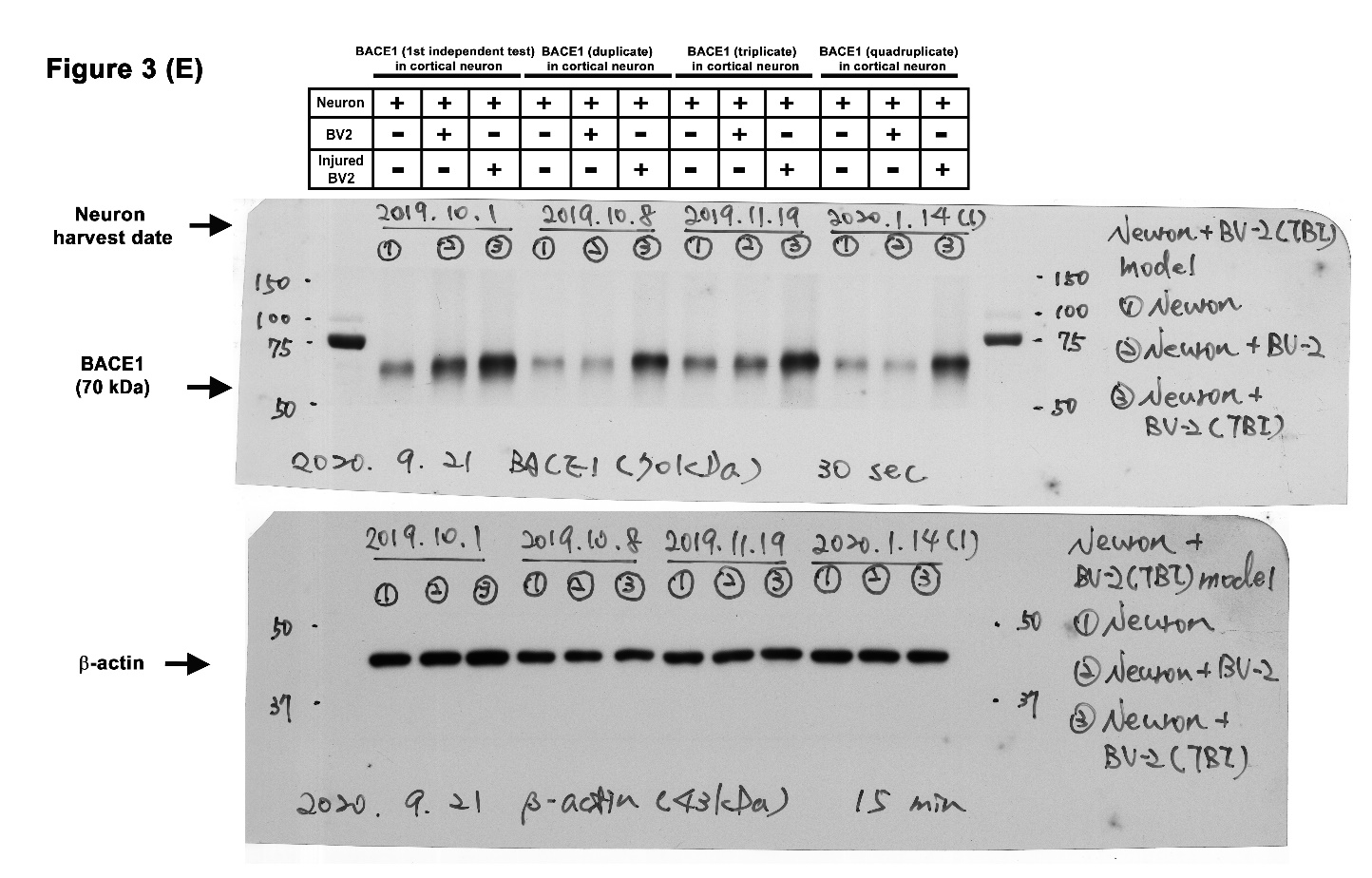


**Supplementary Figure S5:** Original western blots for Figure 3 (E). Labels on the right of each x-film scan indicate the antibody used for detection. Dashed red boxes show the regions that were cropped for the figures.


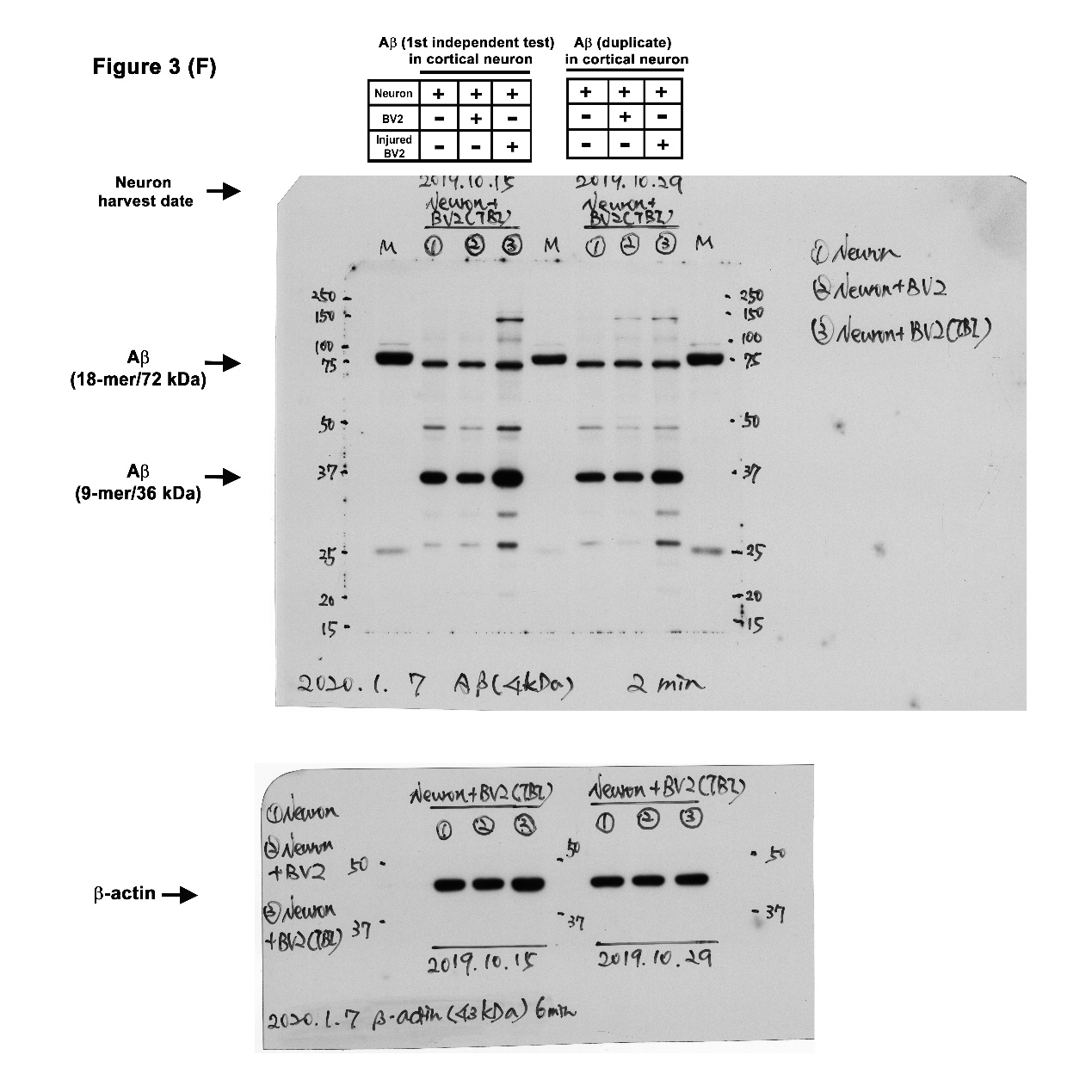


**Supplementary Figure S6:** Original western blots for Figure 3 (F). Labels on the right of each x-film scan indicate the antibody used for detection. Dashed red boxes show the regions that were cropped for the figures.
